# Absorption and distribution of estradiol from male seminal emissions during mating

**DOI:** 10.1530/JOE-16-0247

**Published:** 2016-11-01

**Authors:** Denys deCatanzaro, Tyler Pollock

**Affiliations:** Department of PsychologyNeuroscience & Behaviour, McMaster University, Hamilton, Ontario, Canada

**Keywords:** estrogen, female reproduction, uterus, reproduction, behaviour

## Abstract

Estradiol-17β (E_2_) plays critical roles in female maturation, sexual receptivity, ovulation and fertility. In many mammals, contact with males can similarly affect these female parameters, whereas male excretions contain significant quantities of E_2_. We administered radiolabeled estradiol ([^3^H]E_2_) to male mice in doses representing a small fraction of their endogenous E_2_. These males were paired with sexually receptive females, and radioactivity was traced into the females’ systems. In Experiment 1, males were given [^3^H]E_2_ at 24 and 1 h before mating. Male-to-female [^3^H]E_2_ transfer intensified with increasing numbers of intromissions and spiked in the uterus after insemination. In Experiment 2, sexually experienced young males received [^3^H]E_2_ at 72 and 24 h before mating, and all mated to ejaculation. The copulatory plug deposited in the female reproductive tract contained substantial levels of radioactivity. The uteri, other tissues and blood serum of females displayed radioactivity indicative of E_2_ transfer. In Experiment 3, radioactivity was observed 3 and 18 h after insemination in the females’ uteri and other tissues, including parts of the brain. In Experiment 4, we observed substantial levels of radioactivity in semen as well as the copulatory plugs retrieved from the females after mating. Transferred E_2_ could directly affect abundant estrogen receptors in the female reproductive tract without potential metabolism by the liver. Sexually transferred E_2_ may facilitate uterine preparation for blastocyst implantation. These data converge with several lines of evidence indicating that male-sourced E_2_ can transfer to proximate females in bioactive form, contributing to various mammalian pheromonal effects.

## Introduction

Both endogenous and exogenous estrogens exert powerful influences over mammalian female physiology and behavior. The most potent estrogen is estradiol-17β (E_2_), which affects estrogen receptors (ER) at very low concentrations (Kuiper *et al*. 1997). In juvenile females, E_2_ drives growth and maturation of the reproductive tract (Alonso & Rosenfield 2002). In adult females, E_2_ regulates estrous or menstrual cycling in coordination with progesterone (P_4_) and pituitary gonadotropins. During the follicular phase of the cycle, E_2_ stimulates uterine endometrial growth (Garcia *et al*. 1988) and triggers a mid-cycle surge of luteinizing hormone (LH), which in turn induces ovulation and release of P_4_ during the luteal phase (Butcher *et al*. 1974, Robker & Richards 1998, Freeman 2006). Actions of E_2_ at the hypothalamus can induce female sexual receptivity (Pfaff 1980). After fertilization, E_2_ influences the rate of passage of fertilized ova through the fallopian tubes (Ortiz *et al*. 1979), and small fluctuations in its concentration determine the success or failure of intrauterine implantation of fertilized ova (blastocysts) (Ma *et al*. 2003).

Hormones have generally been assumed to act exclusively within the individual whose glands produce them, but recent data show that some quantity of sex steroids can transfer among cohabiting individuals (Guzzo *et al*. 2012, 2013). Males’ excretions such as urine and perspiration can naturally contain high concentrations of unconjugated, bioactive E_2_ (deCatanzaro *et al*. 2006, 2009, Muir *et al*. 2008). Low molecular mass, polarity and a lipophilic nature permit exogenous E_2_ to be absorbed percutaneously and nasally (Scheuplein *et al*. 1969, Anand Kumar *et al*. 1974, Hueber *et al*. 1994, Guzzo *et al*. 2012). When male mice were injected with a dose of tritiated estradiol ([^3^H]E_2_) equivalent to a fraction of their endogenous E_2_, untreated females that cohabitated with these males for a few days subsequently showed radioactivity in the uterus, ovaries, brain and other tissues (Guzzo *et al*. 2012, 2013). [^3^H]P_4_ can also transfer between individuals, although less readily than does [^3^H]E_2_ (Guzzo *et al*. 2013). Male-to-female [^3^H]E_2_ transfer has also been found in big brown bats, and their great phylogenetic distance from mice suggests that steroid transfer could occur broadly among mammals (deCatanzaro *et al*. 2014). These data satisfy a necessary condition for actions of E_2_ as a ‘pheromone’, if this term is simply defined as a chemical excreted by an individual that can affect the physiology and/or behavior of a proximate conspecific (deCatanzaro 2015).

Indeed, the pertinence of male-to-female transfer of E_2_ has been demonstrated for two well-known mammalian pheromonal effects, male-induced promotion of female puberty (Vandenbergh effect) and novel male-induced blastocyst implantation failure (Bruce effect). Influences of novel male urine on the female nasal area are well established in both effects (Parkes & Bruce 1962, Colby & Vandenbergh 1974, deCatanzaro 2015), and both can be mimicked by giving females minute doses of exogenous E_2_ (Bronson 1975, deCatanzaro *et al*. 1991, 2006). Concentrations of E_2_ in urine of male mice rise when they are proximate to females for a few days, and males actively direct their urine toward females in these circumstances (Beaton *et al*. 2006, deCatanzaro *et al*. 2006, 2009). Castration blocks males’ ability to induce the Vandenbergh and Bruce effects while reducing E_2_ in male urine, whereas restoration of normal urinary E_2_ concentrations via intramuscular E_2_ injections restores their capacity to induce these effects (Thorpe & deCatanzaro 2012). Reduction of urinary E_2_ levels in intact novel males via a drug (anastrozole) that blocks aromatase, which converts testosterone to E_2_, prevents them from inducing the Bruce effect (Beaton & deCatanzaro 2005). Moreover, the effects of novel male exposure on female reproductive maturation and blastocyst implantation failure are highly consistent with known actions of E_2_ on these female reproductive parameters (e.g. Alonso & Rosenfield 2002, Ma *et al*. 2003, Beaton *et al*. 2006, Rajabi *et al*. 2014) and female absorption via nasal and percutaneous means of E_2_ from male excretions such as urine (deCatanzaro 2015).

This study was designed to shed further light on the modes via which E_2_ might transfer between cohabiting individuals. We previously observed substantial radioactivity in testes, epididymides and male accessory glands when males were directly given [^3^H]E_2_, in both mice (Guzzo *et al*. 2012) and bats (deCatanzaro *et al*. 2014). In rodents, herbivores and primates including humans, substantial concentrations of endogenous E_2_ and other estrogens have been found in rete testes fluid, which provides an environment for spermatozoa in the epididymis, and in ejaculated semen (Waites & Einer-Jensen 1974, Eiler & Graves 1977, Free & Jaffe 1979, Reiffsteck *et al*. 1982, Adamopoulos *et al*. 1984, Claus *et al*. 1987, 1992, Lemazurier *et al*. 2002). Apart from some examination of the role of male-sourced E_2_ in uterine contractions in the sow (Claus *et al*. 1987), discussion of adaptive roles of estrogens in the male reproductive tract has focused on functions within the male *per se* (see reviews by Hess 2003, Hess *et al*. 2011), given the presence of ER along the length of the epididymides (Danzo & Eller 1979, Couse *et al*. 1997). We hypothesized that elevated estrogens in male semen may also have evolved due to impacts on the female. Accordingly, we predicted that E_2_ in semen would be absorbed via the female reproductive tract, where there are very high concentrations of ER (Couse *et al*. 1997, Kuiper *et al*. 1997, Hiroi *et al*. 1999). We traced [^3^H]E_2_ given to males, mating them with females and measuring radioactivity in the female’s blood and organs subsequent to mating. In Experiment 1, [^3^H]E_2_ was administered twice to males with varied sexual experience, 24 and 1 h before pairing with females, and transfer occurred in proportion to the progress of mating. In Experiment 2, younger, sexually experienced males received [^3^H]E_2_ earlier (72 and 24 h before mating) to allow greater accumulation in their sexual fluids; this produced pronounced transfer to females, especially via the copulatory plug. In Experiment 3, we observed that E_2_ transferred during mating was still evident in females 3 and 18 h after mating. In Experiment 4, we detected substantial levels of radioactivity in semen retrieved from the uteri of females mated with [^3^H]E_2_-treated males.

## Materials and methods

### Animals

Mice were of CF1 strain obtained from Charles River and were normally housed in standard polypropylene cages measuring 28 × 16 × 11 (height) cm with wire tops allowing continuous access to food and water. The colony was maintained at 21°C with a reversed 14-h light:10-h darkness cycle. This research was approved by the Animal Research Ethics Board of McMaster University, conforming to Canadian Council on Animal Care standards.

### Preparation of sexually receptive females

Females aged 2–3 months were bilaterally ovariectomized under isoflurane anesthesia and allowed to recover for two weeks, then given repeated steroid injections and male exposure following an established procedure that produces full behavioral estrus (deCatanzaro & Gorzalka 1979, deCatanzaro *et al*. 2013). They were first injected sc with 10 µg E_2_ followed 48 h later by 500 µg P_4_, with each steroid in 0.05 mL peanut oil, and then 5 h later, each female was paired with a male for 24 h (pre-exposure procedure). After one week, the steroid injections and male exposure were repeated. Subsequently, the E_2_ and P_4_ injections were repeated, and the female was paired with a male treated with [^3^H]E_2_ as described in the following.

### Experiment 1: Males given [^3^H]E_2_ 24 and 1 h before female exposure

This experiment was designed to produce natural variance in mating across subjects, such that subsets mated to the point of insemination, whereas others showed only mounts or only mounts and intromissions. This was achieved by using older males (aged 8–9 months), some of which had previously been used as breeders for other lines of research, whereas others were sexually inexperienced. Each male received two i.p. injections of 10 µCi ^3^H-E_2_ ([2,4,6,7-[^3^H](N)]-estradiol, in ethanol, 1.0 µCi/µL, 89.2 Ci/mM, PerkinElmer), one at 24 h and the other at 1 h before pairing with females (*n* = 30 male–female pairs). Each injection was equivalent to 30.5 ng E_2_ per male. At approximately 5–6 h after commencement of the darkness phase of the lighting cycle and the female’s P_4_ injection, each male was paired with a receptive female in a 4-L Pyrex beaker with no bedding material in a dimly illuminated room. Paired animals were continuously observed by a trained experimenter for sexual activity, which was measured by standard procedures for this species, counting instances of mounts (without intromission), intromissions (with pelvic thrusting) and ejaculations (McGill 1965, deCatanzaro & Gorzalka 1979, deCatanzaro *et al*. 2013). Observers also recorded any instances of urination and checked the beakers for any signs of urine. The animals remained paired until an ejaculation was observed, or otherwise for a maximum of 2 h. Commencing within 5 min of removal from the female, a random subset of the males (*n* = 14) were anesthetized with isoflurane, blood was collected via cardiac puncture and urine was collected from the bladder. Males were then perfused with 20 mL phosphate-buffered saline (PBS) and dissected. Samples were taken from the olfactory bulb, cerebellum, frontal cortex, hypothalamus (posterior to the optic chiasm and anterior to the pituitary stalk on the ventral surface of the brain), the heart, lung, muscle from the hind leg, abdominal adipose, testes, epididymides, vesicular-coagulating glands, preputial glands, liver and a cross-section of the kidney encompassing both the cortex and the medulla following established methods (Pollock *et al*. 2014, Pollock & deCatanzaro 2014). Approximately 20 min after removal from the males, females were anesthetized in a clean chamber with isoflurane, and blood samples were taken. Each female was perfused with 20 mL PBS, and the uterus was extracted, stripped of adipose and emptied of fluid by pressing it out through a number of incisions. Samples were also taken from the heart, lung, muscle, adipose, liver and kidney. Samples were also taken from the olfactory bulb, cerebellum, frontal cortex and hypothalamus for 10 of the females from this experiment. Each tissue was placed on clean absorbent paper (paper towel) and rolled on it to blot the exterior surface dry. Each sample was placed in a pre-weighed scintillation vial, the vial was re-weighed to determine wet tissue mass and then the tissue was solubilized and measured for radioactivity as described in the following. Beakers for behavioral observation, surgical equipment and work surfaces were decontaminated after every use with Extran MA 01 (EMD Chemicals, Darmstadt, Germany).

### Experiment 2: Sexually experienced young males given [^3^H]E_2_ 72 and 24 h before female exposure

This experiment was designed to examine the distribution of radioactivity in male–female pairs that all efficiently mated to ejaculation, comparing these to two control conditions. The [^3^H]E_2_ injections were given at a longer interval before mating than in Experiment 1 to ensure adequate time for the [^3^H]E_2_ to enter fluids pooled in the preputials, vesicular-coagulating glands and epididymides and to reduce the concentration in the males’ urine. Young, sexually experienced males (aged 90–105 days) were each randomly paired with a female in the pre-exposure procedure before the actual experiment (described previously). In the experimental condition, after one week, the males each received an i.p. injection of 10 µCi [^3^H]E_2_ at 72 h and another such injection at 24 h before random pairing with the females (*n* = 9 male–female pairs). At 5 h, after commencement of the darkness phase of the lighting cycle and P_4_ administration, each male was paired with a receptive female, and animals were continuously observed for sexual activity as described previously. Beginning 5 min after ejaculation and removal from the males, the females were anesthetized with isoflurane, sampled for blood, perfused and dissected. Samples were taken from tissues as described for Experiment 1. The whole copulatory plug was also extracted from the reproductive tract. Approximately 20 min after removal from the females, all males were anesthetized, perfused and dissected to obtain samples as described for Experiment 1. Two control conditions were prepared. In the first control condition (*n* = 8 male–female pairs), males were given vehicle injections instead of [^3^H]E_2_ at 72 and 24 h before pairing with receptive females. After ejaculation, females from these pairs were perfused and dissected, and their tissues were prepared for scintillation counting through the same procedures as the experimental females mated with [^3^H]E_2_-treated males. In the second control condition (*n* = 9 male–female pairs), ovariectomized females were not given any steroids subsequent to surgery, and then were exposed 5 weeks after surgery to males that had been given [^3^H]E_2_ at 72 and 24 h before pairing. The length of exposure to males was matched to the ejaculation latencies of the subjects in the experimental condition, with one female randomly matched to each experimental female.

Additional males (*n* = 5) were each given the [^3^H]E_2_ injections but not mated with females, then sampled for the same tissues at the same interval after the last injection as the female-exposed males in the experimental condition. One whole testis, one epididymis, one vesicular-coagulating gland and one preputial gland were analyzed for these males and compared with samples from the mated males. Tissues were solubilized and measured for radioactivity as described below.

### Experiment 3: Measurement in females 3 and 18 h after mating with males given [^3^H]E_2_

This experiment was designed to determine whether male-sourced E_2_ remains in the female’s system several hours after mating. The procedures replicated those in the experimental condition of Experiment 2, but radioactivity was measured in females 3 h or 18 h after mating. Sexually experienced males aged 115–130 days were injected twice with [^3^H]E_2_, and each was paired with a sexually receptive female as in Experiment 2. All pairs were mated to ejaculation. A random subset of the males (*n* = 4) were killed, perfused and dissected for selected tissues after ejaculation as in the previous experiments. Each female was returned to her home cage after insemination and remained there either for 3 h (*n* = 7) or until the next day approximately 18 h after insemination (*n* = 4). Each female was then anesthetized, sampled for blood, perfused and dissected as in the previous experiments. The same tissues were sampled as in Experiment 2, including the remnants of the copulatory plug where it was found. Brain areas were also sampled, including the olfactory bulb, cerebellum, frontal cortex and hypothalamus.

### Experiment 4: Measurement of [^3^H]E_2_ in ejaculated semen

In conducting the previous experiments, we informally observed that semen could be recovered from the mated female’s uterus. This experiment was designed to determine the levels of [^3^H]E_2_ in semen and compare it to levels in the copulatory plug and the uterus *per se*. Ovariectomized females were prepared and mated to insemination with former proven breeder males (aged 3.5 months) given [^3^H]E_2_ 72 and 24 h before exposure (*n* = 7 male–female pairs). Sexual behavior was continuously observed, but only ejaculation latency was recorded. Beginning at approximately 5 min after insemination, each female was anesthetized and perfused. Serum and the copulatory plug were sampled as described previously. The viscose fluid above the plug and cervix was pressed out and retained in a vial for analysis, and then the uterus was prepared for analysis as described previously.

### Tissue processing for scintillation counting

In all experiments, routine monitoring, involving sampling of all work surfaces and equipment, consistently showed the absence of residual radioactivity, indicating that contamination of samples did not occur. Tissue samples were solubilized by adding 1 mL SOLVABLE (PerkinElmer) to each vial. Vials were placed in a 50°C water bath, agitated after 2 h, and then returned to the bath for 2–3 h until completely dissolved. They were removed and cooled for 10 min, and then 5 mL Ultima Gold Scintillation Cocktail (PerkinElmer) was added to each vial. Vials were agitated again for 10 min to mix the sample and scintillation cocktail. Each vial was stored for 5 min in the darkness chamber of a Tri-Carb 2910 TR Liquid Scintillation Counter (PerkinElmer) to eliminate background heat and luminescence. Radioactivity was then measured for 5 min per vial, and final adjusted estimates for the amount of radioactivity per sample in disintegrations per minute (DPM) were automatically calculated via Quanta-Smart software. The DPM measure was standardized to the weight of the sample wet mass as DPM/mg tissue. Blood samples were prepared by centrifugation at 1500 ***g*** for 10 min, and 10 μL serum was placed in a scintillation vial containing 5 mL Ultima Gold. These vials were agitated for 10 min to mix the sample and scintillation cocktail. Radioactivity was measured as described previously and reported as DPM/µL serum. A concentration of 1 DPM/mg tissue or 1 DPM/µL serum is equivalent to 1.38 pg E_2_/g tissue or 1.38 pg E_2_/mL serum.

### Statistical analysis

DPM values such as those that we report in tissues and fluids are not possible in the sampling distribution of background radiation, which is continually monitored by the scintillation counter and automatically subtracted from each sample. Where possible, analysis of variance was applied, with significant effects followed by pairwise Newman–Keuls multiple comparisons. For female data from Experiment 2, within-subjects (repeated measures) analysis of variance was applied to compare tissues, with multiple comparisons adapted for within-subject analysis as described by Winer *et al*. (1991). Levene’s test indicated that there were highly unequal variances among experimental and control females in Experiment 2, *F* = 9.53, *P* = 0.0001; accordingly these data were analyzed through a conservative nonparametric statistic, the Wilcoxon rank-sum test. In all analyses, the threshold for statistical significance was set at the conventional α level of *P* < 0.05; however, Bonferroni adjustments were applied to the threshold probabilities where multiple tissues were measured within a data set. We report statistics that have associated raw *P* values below the adjusted threshold; for example, where there are four measures, the threshold *P* value for significance is <0.0125.

## Results

### Experiment 1: Distribution in females relates to degree of sexual behavior with males given [^3^H]E_2_ 24 and 1 h before mating

**Figure 1:**
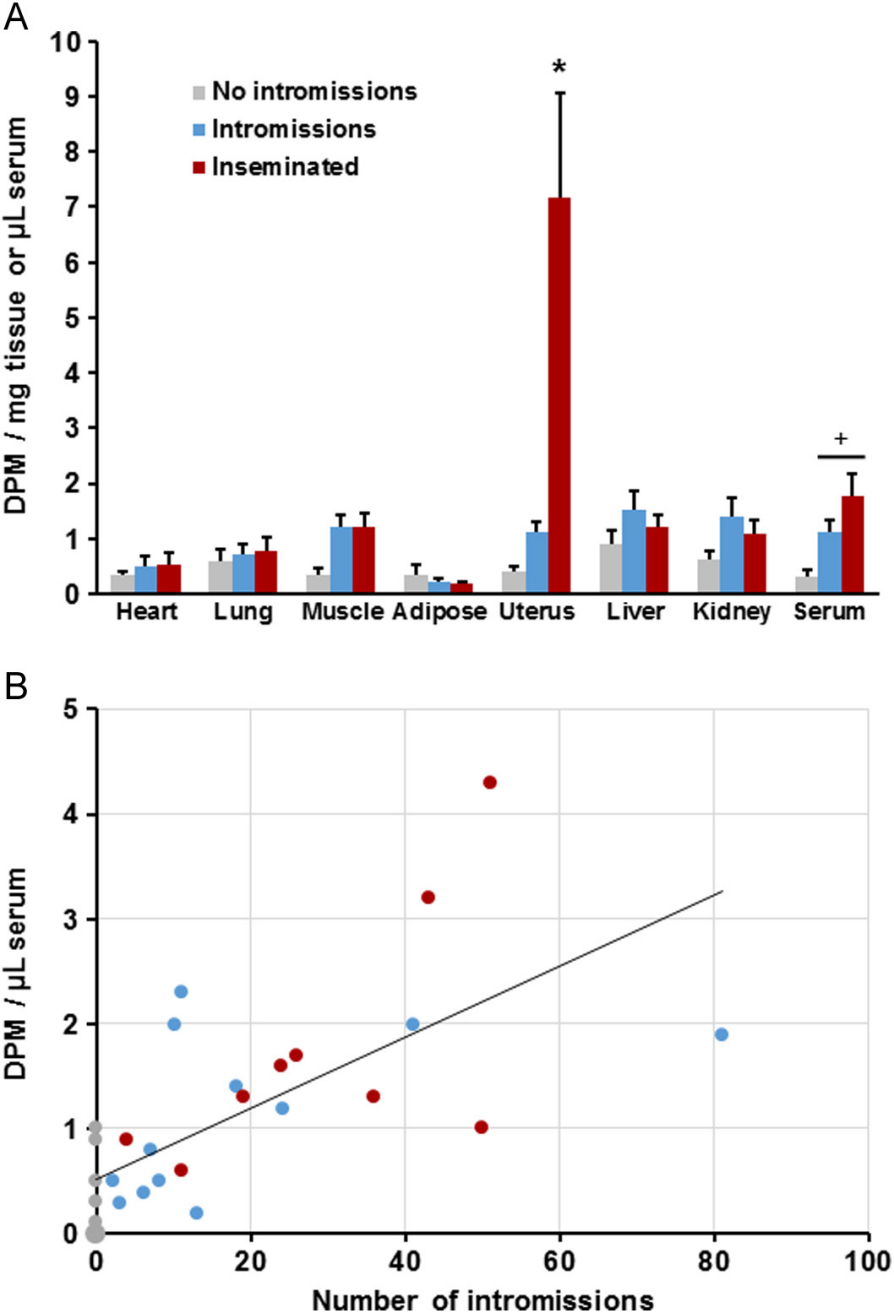
(A) In Experiment 1, the mean + s.e.m. radioactivity in serum and tissues taken from females after mating with males given [^3^H]E_2_ at 24 and 1 h before mating. Three categories of females are shown: those that did not receive any intromissions (No intromissions, *n* = 9), those that received intromissions but no ejaculation (Intromissions, *n* = 12) and those that received an ejaculation (Inseminated, *n* = 9). *indicates that radioactivity in this tissue in inseminated females significantly exceeded that in each of the other categories; ^+^indicates that females in each of these categories showed significantly more radioactivity than those that did not receive any intromissions. (B) Scatterplot and linear regression line relating the number of intromissions observed and radioactivity subsequently measured in female serum. The marker at zero intromissions and zero DPM represents four females; each other marker represents one female. A full colour version of this figure is available at http://dx.doi.org/10.1530/JOE-16-0247.

Radioactivity was widely distributed in the tissues of males directly given ^3^H-E_2_, including the testes, epididymides, vesicular-coagulating glands and preputial glands (Table 1). The greatest mean levels in males were observed in urine, liver and serum. Descriptive statistics for sexual behavior are given (Table 2). Within the 2-h session, 9 males failed to show any intromissions, 12 showed intromissions but did not ejaculate and 9 mated to insemination. Females were divided into these three categories, and radioactivity in their serum and tissues was analyzed (Fig. 1A). There was a significant effect of category in the measure of radioactivity in the uterus, *F*(2,27) = 9.18, *P* = 0.001; multiple comparisons indicated that radioactivity in the females that were inseminated exceeded that of females in each of the other two categories. There was also a significant effect of category in serum, *F*(2,27) = 6.78, *P* = 0.004; multiple comparisons indicated that both the females that received intromissions and those that were inseminated exceed those that did not receive any intromissions. A scatterplot relating number of intromissions to radioactivity in serum is given (Fig. 1B), and the linear correlation was significant, *r* = 0.683, *t*(28) = 4.95, *P* = 0.0001. The 10 females whose brain tissues were measured showed mean ± s.e.m. DPM/mg for the olfactory bulb, cerebellum, frontal cortex and hypothalamus for the three categories were respectively as follows: females subject to no intromissions (*n* = 4), 0.12 ± 0.12, 0.23 ± 0.13, 0.20 ± 0.08, 0.02 ± 0.02; females receiving intromissions but not inseminated (*n* = 4), 0.08 ± 0.24, 0.59 ± 0.33, 0.73 ± 0.30, 0.83 ± 0.43 and females that were inseminated (*n* = 2), 1.45 ± 0.38, 1.47 ± 0.72, 0.99 ± 0.65, 1.42 ± 0.82.

**Table 1:** Radioactivity in subsets of [^3^H]E_2_-treated males measured after mating in Experiments 1 (*n* = 14), 2 (*n* = 9) and 3 (*n* = 4). In Experiment 1, [^3^H]E_2_ was given to males 24 and 1 h before exposure to females. In Experiments 2 and 3, [^3^H]E_2_ was given to males 72 and 24 h before exposure to females. Tissue sampling commenced within 20 min after removal from females.

	**Experiment 1**	**Experiment 2**	**Experiment 3**
Olfactory bulb	170 ± 17	205 ± 20	241 ± 35
Cerebellum	117 ± 10	152 ± 20	206 ± 39
Frontal cortex	178 ± 18	237 ± 36	218 ± 32
Hypothalamus	163 ± 11	198 ± 27	191 ± 33
Heart	91 ± 8	93 ± 8	86 ± 21
Lung	281 ± 97	139 ± 17	122 ± 46
Muscle	263 ± 15	237 ± 34	287 ± 28
Adipose	105 ± 24	55 ± 20	34 ± 6
Testes	308 ± 15	258 ± 14	336 ± 32
Epididymides	277 ± 16	213 ± 12	261 ± 16
Vesicular-coagulating glands	260 ± 18	195 ± 15	253 ± 24
Preputial glands	121 ± 13	131 ± 9	158 ± 15
Liver	641 ± 103	271 ± 44	337 ± 34
Kidney	160 ± 16	144 ± 23	201 ± 40
Serum	406 ± 14	340 ± 13	354 ± 61
Urine	3042 ± 435	443 ± 32	743 ± 243

The values are mean ± s.e.m. DPM/mg for tissues and DPM/µL for serum and urine.

**Table 2:** Sexual activity in pairs with [^3^H]E_2_-treated males in Experiments 1, 2 and 3. The measures are mean ± s.e.m. counts of the number of mounts, intromissions and ejaculations, and latencies to the first of each such response from session commencement. In Experiment 1, [^3^H]E_2_ was given to males 24 and 1 h before exposure to females, and some pairs did not show intromissions (No intromissions, *n* = 9), others did so but without insemination (Intromissions, *n* = 12), whereas others showed an ejaculation (Inseminated, *n* = 9). In Experiments 2 (*n* = 9) and 3 (*n* = 11), [^3^H]E_2_ was given to males 72 and 24 h before exposure to females and all pairs mated to insemination.

	**Experiment 1**				
	**No Intromissions**	**Intromissions**	**Inseminated**	**Experiment 2**	**Experiment 3**
Number of mounts	17.0 ± 6.8	42.0 ± 9.5	20.7 ± 5.3	21.7 ± 7.2	28.3 ± 11.0
Number of intromissions	0.0 ± 0.0	19.0 ± 6.5	29.2 ± 5.6	30.9 ± 10.3	21.4 ± 4.4
Number of ejaculations	0.0 ± 0.0	0.0 ± 0.0	1.0 ± 0.0	1.0 ± 0.0	1.0 ± 0.0
Mount latency (min)	75.0 ± 18.0	19.0 ± 5.7	23.1 ± 11.4	9.0 ± 3.0	12.6 ± 3.7
Intromission latency (min)	n/a	42.0 ± 6.0	26.6 ± 11.0	16.2 ± 5.4	26.8 ± 6.5
Ejaculation latency (min)	n/a	n/a	70.8 ± 13.3	52.9 ± 17.6	89.4 ± 14.8

### Experiment 2: Distribution in inseminated females exposed to males given [^3^H]E_2_ 72 and 24 h before mating

**Figure 2:**
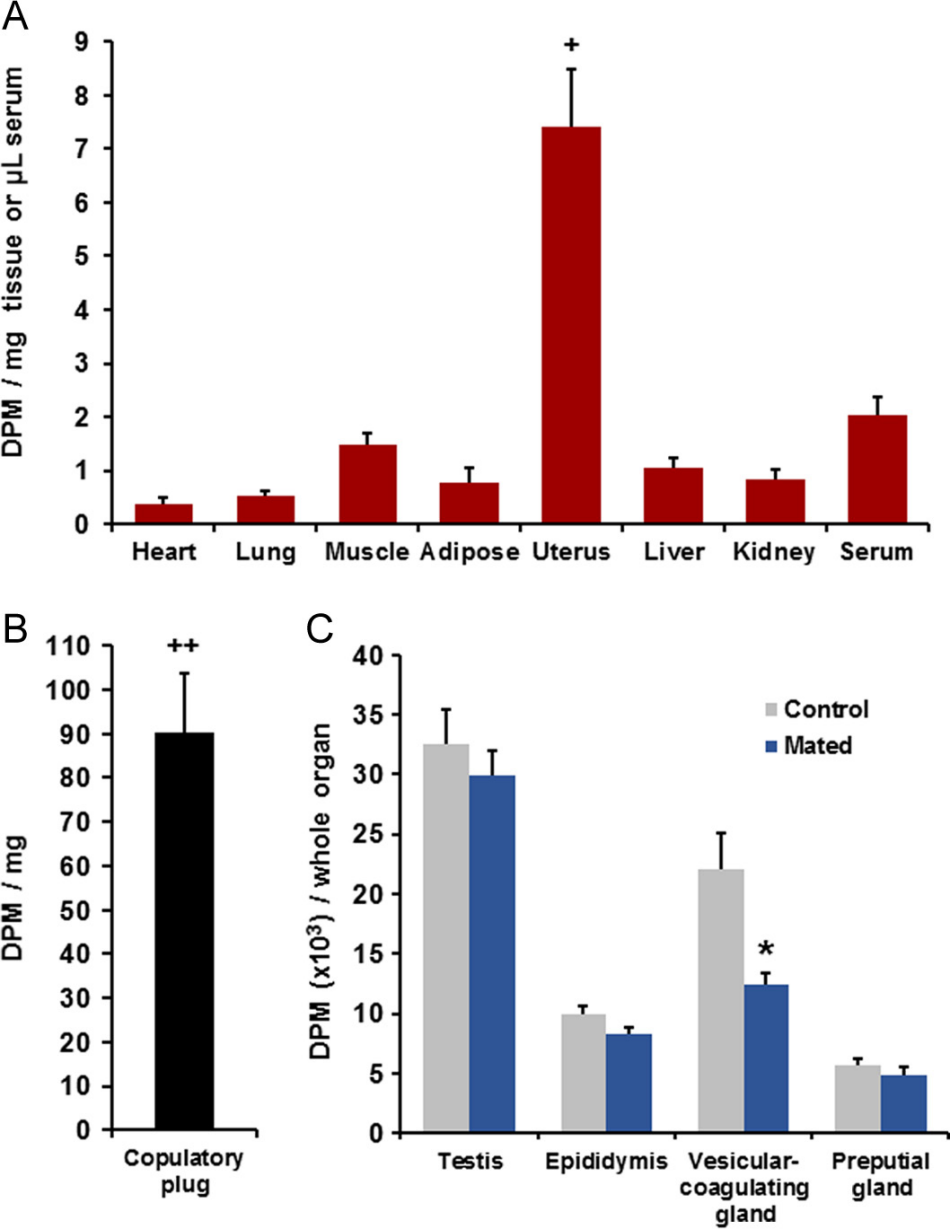
(A) In Experiment 2, the mean + s.e.m. radioactivity in serum and tissues taken from females (*n* = 9) after mating with young sexually experienced males given [^3^H]E_2_ at 72 and 24 h before mating. All males mated to ejaculation. ^+^indicates that radioactivity in this measure significantly exceeded that in all other female tissues, *P* < 0.01. There were also two control conditions (see Table 3), females that were exposed to males given vehicle injections without [^3^H]E_2_ and ovariectomized females that were not given replacement E_2_ and P_4_, and then exposed to [^3^H]E_2_-treated males; both showed values at or near zero DPM in every measure. (B) Radioactivity in the copulatory plugs retrieved from the vaginal canal in this experiment (*n* = 9). ^++^indicates that radioactivity in the plug significantly exceeded that for all female tissues, *P* < 0.01. (C) Radioactivity in one whole testis, epididymis, vesicular-coagulating gland and preputial gland from mated males (*n* = 9) and from unmated control males given [^3^H]E_2_ at the same times before measurement (*n* = 5). *denotes a significant difference between the mated and unmated males, *P* < 0.05. A full colour version of this figure is available at http://dx.doi.org/10.1530/JOE-16-0247.

All pairs mated to ejaculation. Radioactivity was widely distributed among the organs and fluids of the males (Table 1). Descriptive statistics for sexual behavior are given (Table 2). Radioactivity was also found in the females’ serum and tissues (Fig. 2A). The copulatory plugs taken from the females’ reproductive tracts contained high levels of radioactivity comparable with those of male tissues (Fig. 2B). Within-subjects analysis of variance comparing samples from females including the copulatory plugs showed significance, *F*(7,56) = 43.62, *P* < 0.0001. Multiple comparisons showed that values for the copulatory plugs significantly exceeded those of all endogenous female tissues, and the values for the uterus significantly exceeded those of all other female tissues. Radioactivity values (DPM/mg tissue or DPM/µL serum) in the two control conditions are shown in Table 3. Females that were exposed to males given vehicle injections (control condition 1) showed 0.000 DPM for all subjects in all tissues and serum, except one subject that showed 0.102 DPM/mg in the kidney and another subject that showed 0.036 DPM/mg in the copulatory plug. The individual DPM values for subjects in this condition were in completely non-overlapping ranges from those in females exposed to [^3^H]E_2_-treated males (experimental condition, Fig. 2A and B) for each tissue, except the heart and adipose where some females exposed to [^3^H]E_2_-treated males showed 0.000 DPM. The Wilcoxon rank-sum test was significant for the lung, muscle, uterus, liver, kidney, serum and copulatory plug; in each case, *W*_s_ = 36, *P* < 0.001. Females that were not given replacement E_2_ and P_4_ after ovariectomy (control condition 2) were mounted by males in 5 of the 9 pairs (mean = 9.0 ± 4.3 mounts), but females resisted mating and no intromissions were observed. Some urine was seen in 4 of the 9 chambers and males were all observed to groom females. The reproductive tracts of these females all were observed to have atrophied. These females showed 0.000 DPM in all measures, except for one female that showed 0.114 DPM/mg in the kidney. The Wilcoxon rank-sum test comparing control condition 2 to the experimental condition showed significance for all measures, with *W*_s_ = 27, except the kidney where *W*_s_ = 31; *P* < 0.001 for all comparisons.

**Table 3:** Radioactivity in the two control conditions among females exposed to males given injections 72 and 24 h before mating in Experiment 2. Control 1 (*n* = 8) involved ovariectomized females made receptive through injections of E_2_ and P_4_ that mated to ejaculation with males given vehicle injections that did not contain [^3^H]E_2_. Control 2 (*n* = 9) involved ovariectomized females that were not given replacement E_2_ and P_4_, paired with males given [^3^H]E_2_, with the durations of pairing matched to those of experimental females.

	**Control 1**	**Control 2**
Heart	0.000 ± 0.000	0.000 ± 0.000
Lung	0.000 ± 0.000	0.000 ± 0.000
Muscle	0.000 ± 0.000	0.000 ± 0.000
Adipose	0.000 ± 0.000	0.000 ± 0.000
Uterus	0.000 ± 0.000	0.000 ± 0.000
Liver	0.000 ± 0.000	0.000 ± 0.000
Kidney	0.013 ± 0.013	0.013 ± 0.013
Serum	0.000 ± 0.000	0.000 ± 0.000
Copulatory plug	0.004 ± 0.004	n/a

The values are mean ± s.e.m. DPM/mg for tissues and DPM/µL for serum.

Thus, the [^3^H]E_2_-treated males in the experimental condition deposited substantial amounts of [^3^H]E_2_ into the females’ reproductive tracts, and some of this diffused into their circulation and bodily organs. Radioactivity in reproductive organs of mated males was compared with that of unmated males that were measured at the same interval after [^3^H]E_2_ treatment (Fig. 2C). Mating induced a significant (one-tailed) reduction of [^3^H]E_2_ in the vesicular-coagulating gland *t*(12) = 3.65, *P* = 0.002. There was also a trend in the epididymis, *t*(12) = 2.01, *P* = 0.033, which was not below the Bonferroni-adjusted threshold.

### Experiment 3: Distribution in females 3 and 18 h after mating with males given [^3^H]E_2_ 72 and 24 h before mating

**Figure 3:**
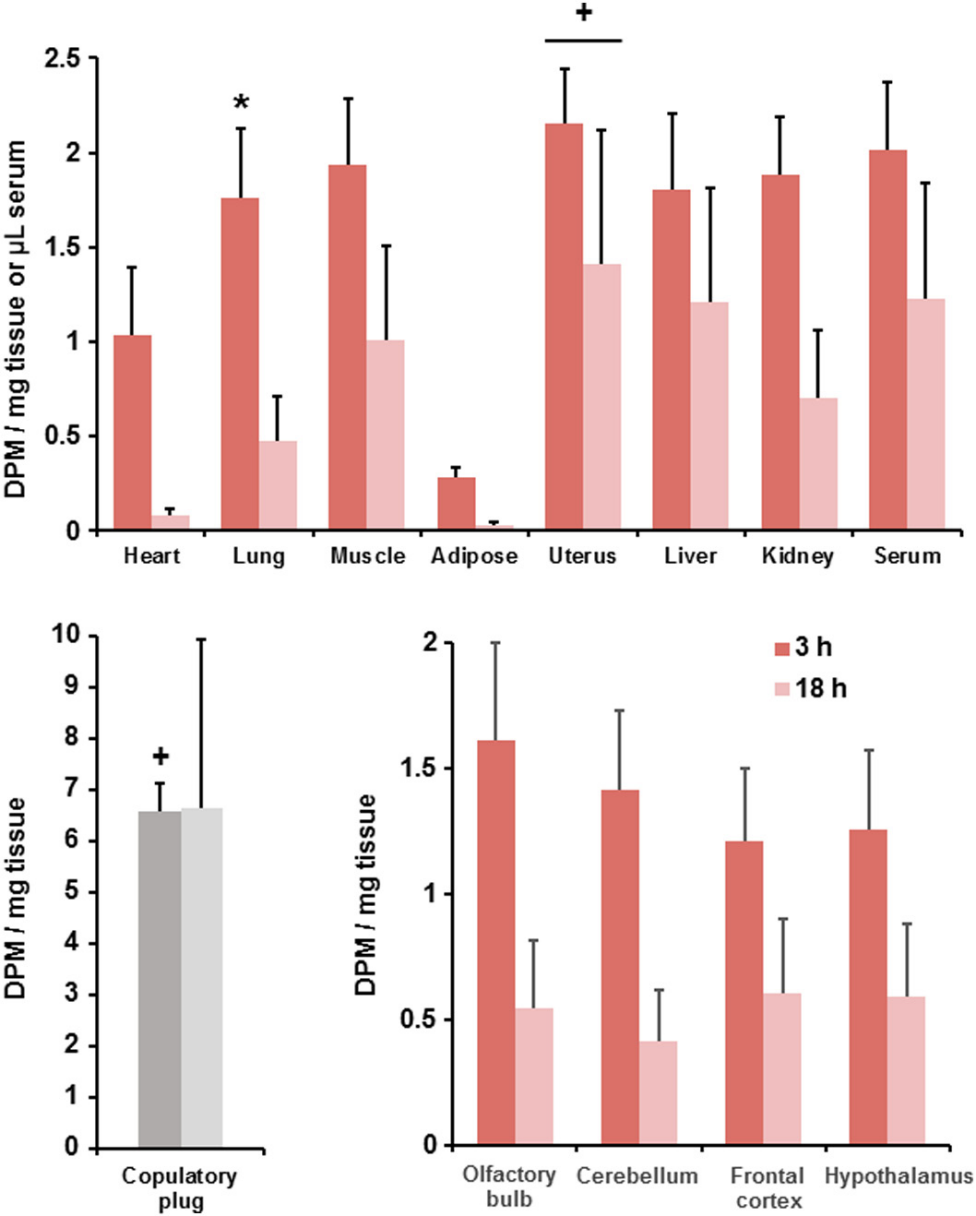
In Experiment 3, the mean + s.e.m. radioactivity in serum and tissues of females measured at 3 h (*n* = 7) or 18 h (*n* = 4) after mating with males given [^3^H]E_2_ at 72 and 24 h before mating. Data are from individual females paired with males that mated to ejaculation. *indicates that the radioactivity exceeds the measures at 0 h (Fig. 2) and 18 h after sexual behavior. ^+^indicates that the radioactivity is significantly lower than at 0 h after sexual behavior (Fig. 2). Note that copulatory plug remnants were only detected for two females in samples measured 18 h after sexual behavior and that these data were excluded from statistical analysis. A full colour version of this figure is available at http://dx.doi.org/10.1530/JOE-16-0247.

Radioactivity was broadly dispersed among males’ tissues (Table 1). Descriptive statistics for sexual behavior are given (Table 2). When females’ tissues were sampled 3 or 18 h after insemination, radioactivity clearly remained in the serum and tissues of each female, including brain and peripheral tissues (Fig. 3). Values at the 3-h interval were similar to or greater than those taken directly after insemination (Fig. 2) for serum and tissues other than the uterus. Analysis of variance comparing female samples at 0, 3 and 18 h after insemination showed significance for the lung, *F*(2,17) = 8.60, *P* = 0.003; and the uterus, *F*(2,17) = 14.59, *P* < 0.001. Multiple comparisons for the lung indicated that radioactivity in the 3-h condition exceeded that at each of the 0-h and 18-h conditions. Multiple comparisons for the uterus indicated that radioactivity in the 0-h condition exceeded that at each of the 3-h and 18-h conditions. The copulatory plug was still found in all the females at 3 h, and remnants were found in two of the four females at 18 h after insemination, but radioactivity was greatly reduced compared with plugs taken directly after insemination (Fig. 2). Statistical comparison showed that radioactivity at 0 h after insemination was significantly greater than that at 3 h after insemination, *t*(14) = 5.42, *P* < 0.001.

### Experiment 4: Radioactivity in ejaculated semen

**Figure 4:**
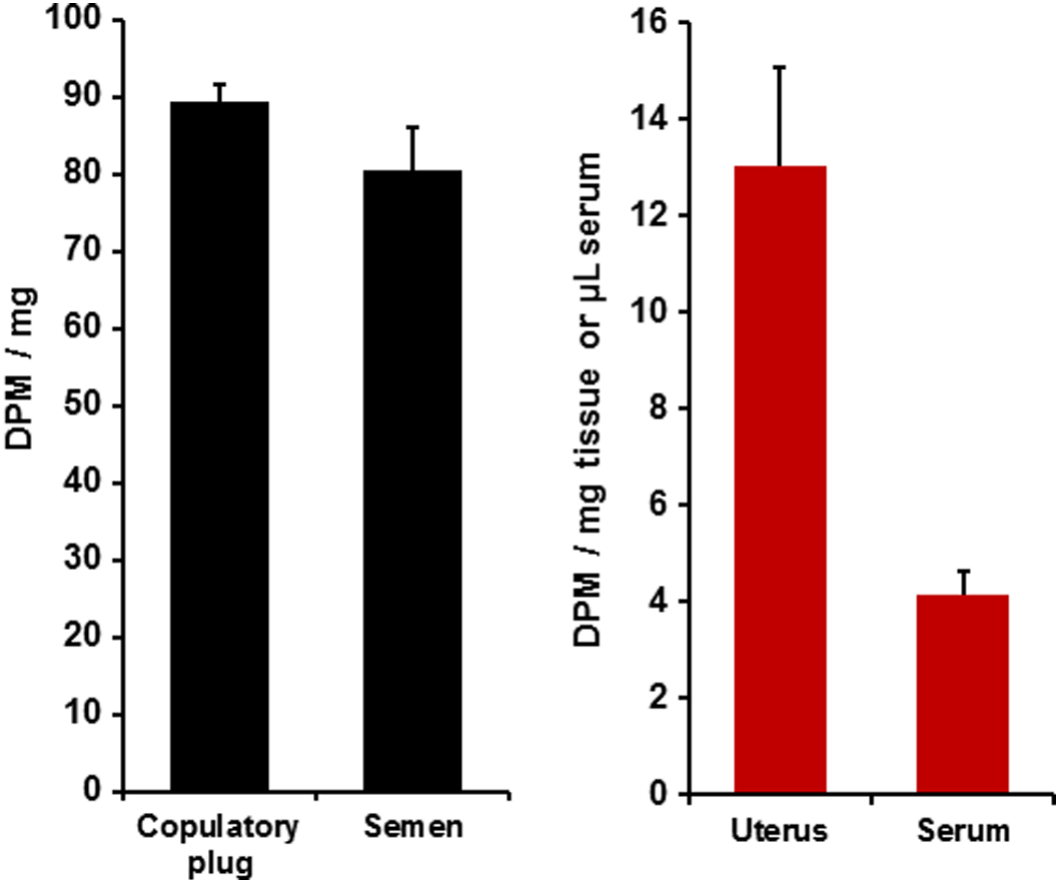
In Experiment 4, the mean + s.e.m. radioactivity in the copulatory plugs and semen recovered from females’ reproductive tracts after insemination, and the serum and uterus of females after mating with males given [^3^H]E_2_ at 72 and 24 h before mating (*n* = 7). A full colour version of this figure is available via http://dx.doi.org/10.1530/JOE-16-0247.

All pairs mated to insemination, with a mean ejaculation latency of 168.4 ± 21.3 min. The semen was readily collected from the females’ uteri and showed substantial levels of radioactivity very similar to those in the copulatory plug (Fig. 4). The range among subjects in semen was 21.3–136.9 DPM/mg. This likely included some fluids from the females themselves, which would mean that the radioactive content of semen is underestimated. The range in the copulatory plugs was 66.4–108.5 DPM/mg.

## Discussion

These experiments demonstrate that E_2_ rapidly transfers from males to females during sexual activity via vaginal absorption from semen, the copulatory plugs and other male emissions. Male-to-female E_2_ transfer began very early in mating and intensified with increasing numbers of intromissions. E_2_ transfer spiked at ejaculation, as a high concentration of E_2_ was deposited directly into the female reproductive tract. This left detectable radioactivity in the uterus, brain and other tissues of females for at least k18 h after mating.

Measurements of radioactive transfer in these experiments significantly underestimate natural transfer of E_2_ during mating. Estimates suggest that the exogenous [^3^H]E_2_ given to males represented just a fraction of their endogenous E_2_, as measures of urinary radioactivity after 10 µCi [^3^H]E_2_ injection in intact males correspond to urinary E_2_ levels that are significantly below natural concentrations (Guzzo *et al*. 2013). Furthermore, the total of 61 ng of E_2_ given to each stimulus male in the form of [^3^H]E_2_ in this study is substantially lower than the dose of E_2_ necessary to maintain normal urinary E_2_ concentrations in castrated male mice, which was 60 µg every other day (Thorpe & deCatanzaro 2012). Also, we only permitted mating to a maximum of one ejaculation, whereas pairs of mice that mate freely often exhibit two or more ejaculations when the female is in estrus (McGill 1965, Mosig & Dewsbury 1976).

Previous work showing pheromonal influences of male-to-female E_2_ transfer indicated that urine was the primary vector (deCatanzaro *et al*. 2006, 2009, Thorpe & deCatanzaro 2012, deCatanzaro 2015). In the current research, we never observed any urination during mating of experimental animals; mouse urine is conspicuous in glass beakers without bedding, and we carefully observed each pair for any instances. Accordingly, it is unlikely that urine was the medium for E_2_ transfer during mating. Also, the reduction of radioactivity in the epididymides and vesicular-coagulating glands in mated males of Experiment 2, and the high radioactivity levels in the transferred semen and copulatory plugs, indicate that fluids transferred during insemination are critical. However, the fact that some radioactivity transferred without ejaculation suggests that other pre-ejaculatory male excretions can carry some E_2_ to the female. Mating male mice typically display oral contact with the females’ genital region during the early phases of mating, and some fluids from male sex-accessory glands will enter the female vaginal canal during mounting and intromission. Data from receptive females with intact uteri in Experiment 1 did show some absorption of radioactivity from males when there were mounts and/or intromissions without insemination. This contrasts with control condition 2 in Experiment 2, where there was almost no transfer to nonreceptive females whose reproductive tracts had atrophied after ovariectomy without hormone replacement, despite some mounting by [^3^H]E_2_-treated males. This contrast underscores the importance of vaginal transfer in pre-ejaculatory copulation.

The ejaculate clearly provides the greatest quantity of E_2_ to the female reproductive tract. Moreover, the copulatory plug provides a bolus in the female’s system that can release E_2_ for some time after ejaculation. The data from Experiment 3 indicate that most of the E_2_ in the copulatory plug had been absorbed into the female’s system within 3 h after insemination, although the plug remained evident as a source of additional E_2_ in half of the females examined after 18 h. This locally deposited E_2_ is proximate to the particularly abundant ER of the female reproductive tract (Couse *et al*. 1997), and it may reach these receptors without being subjected to enzymes in the liver and elsewhere that might conjugate the hormone. Sexually received E_2_ can clearly also be absorbed into blood circulation, from which it can pass to organs throughout the body. As seen in Experiment 3, this includes the brain, where there are concentrations of receptors in areas such as the ventromedial hypothalamus (Pfaff 1980, Couse *et al*. 1997).

We demonstrated here that E_2_ is transferred to females throughout mating, but especially during insemination. Our data suggest that effects of such male-sourced E_2_ are most likely to occur in the uterus, and they may account for some dynamics that have previously been attributed to the female’s endogenous E_2_ (Dey *et al*. 2004). It is established that the proliferation and differentiation of uterine cells in preparation for blastocyst implantation is regulated by coordinated actions of E_2_ and P_4_ (Huet-Hudson *et al*. 1989, Dey *et al*. 2004). Subsequent to insemination and before blastocyst arrival in the uterus, E_2_ binds to the uterus and promotes epithelial cell proliferation. E_2_ has inflammatory effects in the uterus, causing tissue edema and induction of P_4_ receptors, while promoting the influx of leukocytes including neutrophils, eosinophils, macrophages and uterine natural killer cells (Tibbetts *et al*. 1999, Hunt *et al*. 2000). E_2_ also facilitates transport of preimplantation embryos from the oviduct to the uterus (Roblero & Garavagno 1979). Receptivity of the uterus in mice also requires a small amount of E_2_ after P_4_ priming (Huet-Hudson & Dey 1990).

Male-sourced uterine E_2_ can also end a pregnancy sired by a previous male. Small elevations of E_2_ above optimal levels, mimicked by as little as 10 ng systemically from an exogenous source, can terminate blastocyst implantation (Ma *et al*. 2003). This has been studied in the context of the Bruce effect, which has been increasingly linked to E_2_ levels in urine and other excretions of novel males (deCatanzaro *et al*. 2006, Guzzo *et al*. 2012, Thorpe & deCatanzaro 2012, deCatanzaro 2015). Exogenous E_2_ can cause premature embryo arrival at the uterus (Ortiz *et al*. 1979), impede uterine closure and blastocyst adhesion to the uterine epithelium via actions on the protein e-cadherin (Rajabi *et al*. 2014), and adversely affect blastocyst development (Valbuena *et al*. 2001). When novel males can sexually interact with previously inseminated females during the peri-implantation period, the pregnancy is often replaced (deCatanzaro *et al*. 1996) and the probability of loss of the initial pregnancy correlates with the number of intromissions by the novel male (deCatanzaro & Storey 1989). The effect of E_2_ transferred from the novel male to the previously inseminated female may terminate implantation of blastocysts sired by the previous male and thereby reset the window of implantation to favor ova fertilized by the novel male. The bolus of E_2_ from the copulatory plug could stimulate ER in the reproductive tract, even when the total quantity of transferred E_2_ is much less than the threshold systemic dose at which implantation fails (cf. deCatanzaro *et al*. 1991, 2001, Ma *et al*. 2003).

One caveat is that measures of radioactivity may not entirely reflect unconjugated E_2_. However, unlike the situation in some other mammals, conjugates of E_2_ have been found to be scarce in excretions of female mice and undetectable in those of male mice, whereas unconjugated E_2_ is abundant in their urine (Muir *et al*. 2001). As indicated previously, unconjugated E_2_ is present in the semen of several mammals. Measures in this study consistently indicate that radioactivity is greatest in the uterus, where ER are much more abundant than in other female tissues. Moreover, as mentioned, E_2_ from the copulatory plug could directly access the uterus without passage through the liver. Exogenous E_2_ given to females remains largely in unconjugated form after various modes of delivery (Schaefer *et al*. 1982, Bawarshi-Nassar *et al*. 1989) and induces effects known to be associated with bioactive estrogens, such as promotion of uterine growth (Hisaw 1959), induction of sexual receptivity after ovariectomy as in female subjects in the current work or disruption of blastocyst implantation (Rajabi *et al*. 2014). Castrated males administered E_2_ intramuscularly can induce implantation failure in inseminated females and uterine growth in juvenile females, unlike vehicle-treated castrates, indicating estrogenic activity despite the exogenous E_2_ being potentially subjected to metabolism in both the male and females (Thorpe & deCatanzaro 2012). Pre-administration of unlabeled E_2_ to females significantly diminishes radioactivity in their uteri after cohabitation with [^3^H]E_2_-treated conspecifics for a few days, consistent with binding of the [^3^H]E_2_ at uterine receptors (Guzzo *et al*. 2013).

Although less likely than direct actions at the uterus, pheromonal impacts of sexually transmitted E_2_ might also occur due to its absorption into blood circulation and interaction with ER in other tissues, especially parts of the brain. Our data show the presence of E_2_ in blood serum and brain tissues for many hours after insemination. Even where males may mount nonreceptive or juvenile females without ejaculation, male-to-female transfer of E_2_ could help advance the development of the female reproductive tract and sexual receptivity. Very low concentrations of E_2_ in the ventromedial hypothalamus are established to have strong influences over female sexual response (Pfaff 1980). Hypothalamic GnRH release is stimulated by kisspeptin, which in turn is stimulated by E_2_ (Maeda *et al*. 2007, Kenealy & Terasawa 2011), which can, in addition to uterine actions of E_2_ (Alonso & Rosenfield 2002, Thorpe & deCatanzaro 2012), contribute to female pubertal development. By stimulating luteinizing hormone release from the anterior pituitary, exogenous E_2_ can also promote ovulation (Karsch *et al*. 1973, Maeda *et al*. 2007) and stimulate P_4_ release (Freeman 2006). In species like humans where mating occurs throughout the menstrual/estrous cycle, multiple matings over the cycle may mean that a considerable amount of E_2_ could be transferred. Considering many established pheromonal influences of adult male exposure on female reproductive states in diverse mammals (deCatanzaro 2015), the current data support the notion that male mammals may have evolved to promote their own reproduction by dosing females with what is arguably their most powerful hormone, E_2_.

## Declaration of interest

The authors declare that there is no conflict of interest that could be perceived as prejudicing the impartiality of the research reported.

## Funding

This research was supported by grants (RGPIN/1199-2010, RGPIN/03649-2015 and EQPEQ/390407-2010) to D deC from the Natural Sciences and Engineering Research Council of Canada (NSERC).
